# Non‑pharmacological care for early-stage dementia through smart environments in Colombia: a mixed‑methods study and methodological guide for caregivers and patients

**DOI:** 10.12688/f1000research.177177.1

**Published:** 2026-03-25

**Authors:** Mariano Romero-Torres, Jon Arambarri, Tobias A. Parodi-Camano

**Affiliations:** 1 Corporacion Unificada Nacional de Educacion Superior, Bogotá, Bogota, Colombia; 2 Universidad Nacional Abierta y a Distancia, Bogotá, Bogota, Colombia; 3 Universidad Internacional Iberoamericana, Campeche, Campeche, Mexico; 4 Universidad de Cordoba, Montería, Cordoba, Colombia

**Keywords:** caregivers, Colombia, dementia, non-pharmacological care, smart environments

## Abstract

**Background:**

Dementia is increasing in Latin America, creating demand for non-pharmacological support that can be delivered safely at home. Smart environments and related digital tools may help caregivers and people with early-stage dementia by supporting safety, reminders, and communication. This study assessed needs and acceptability in Colombia and produced a methodological guide for technology selection.

**Methods:**

We conducted a sequential exploratory mixed-methods study. First, a focused evidence synthesis informed a feature catalogue and instrument design. Second, we administered a cross-sectional questionnaire to caregivers and people living with early-stage dementia. Quantitative data were summarised with descriptive statistics and non-parametric group comparisons; open-ended responses were analysed thematically and integrated with the quantitative findings.

**Results:**

Fifty-one responses were analysed. Safety-oriented functions (for example, fall detection and geolocation), reminders for activities of daily living, tele-assistance, and cognitive tele-stimulation were the most frequently prioritised. Acceptability was generally higher for low-burden technologies with clear usefulness, and age differences were limited across key comparisons.

**Conclusions:**

In this sample, smart-environment-enabled non-pharmacological support was feasible and broadly acceptable for early-stage dementia care. The methodological guide emphasises prioritising safety and reminders, reducing interaction burden, and incorporating privacy-by-design. Further studies should validate these findings with larger and more diverse samples and evaluate implementation outcomes.

## Introduction

Dementia imposes substantial cognitive, psychosocial, and economic burdens on patients, families, and health systems ( World Health Organization, 2012; Prince et al., 2015; Wimo et al., 2017; Livingston et al., 2017). Non-pharmacological interventions supported by assistive technologies and smart environments can preserve autonomy, reduce caregiver strain, and enable timely, person-centered support. Building on prior work on technology-enabled care and regional needs in Colombia, we aimed to generate context-specific evidence and consolidate it into a practical methodological guide.

From a theoretical perspective, senile dementia is recognized by the World Health Organization ( World Health Organization, 2025) as a chronic and progressive neurodegenerative disorder that impairs memory, reasoning, and communication, often accompanied by emotional and behavioral alterations. In Latin American contexts, including Colombia, the care of people with dementia largely falls on informal caregivers, mainly family members, who experience high levels of physical and emotional stress ( Ramírez, 2018). This dynamic highlights the urgent need to design comprehensive, culturally sensitive, and technologically assisted models of care.

Theoretical frameworks derived from recent doctoral research in Colombia ( Romero-Torres, 2025) emphasize that the integration of smart environments and assistive technologies—such as teleassistance, telestimulation, movement-based systems, and audio-based or robotic aids—can improve quality of life by enhancing autonomy and reducing caregiver dependency. These interventions, when designed under a non-pharmacological therapeutic model, support the maintenance of cognitive and social skills while fostering emotional balance in early-stage dementia. Moreover, they align with the concept of *humanized digital transformation*, where technology complements, rather than replaces, the caregiver’s role.

Global literature corroborates these premises. Kiselica et al. (2024) propose the CARES model—which includes cognitive offloading, automation, remote monitoring, emotional/social support, and symptom management—as a conceptual basis for technology-assisted dementia care. Similarly, Löbe and AboJabel (2022) demonstrate that intelligent assistive technology (IAT) can empower individuals with mild or moderate dementia to live independently for longer. Studies such as De Oliveira (2023) and Val and Cardoso (2021) reinforce that the ethical use of assistive technologies promotes empathy, reduces costs, and improves both patient and caregiver well-being.

In Colombia, these findings converge with national needs. A community-based dementia care organization in the Caribbean region of the country provides a relevant context for applying these approaches. The lack of specialized services in geriatric mental health and the social stigma surrounding cognitive decline make the implementation of accessible, user-centered technological solutions a public health priority. Therefore, this study not only addresses a scientific gap but also responds to a social demand for adaptable, inclusive, and sustainable care models.

## Research hypotheses

Based on the theoretical framework of Romero-Torres (2025) and complementary empirical studies ( Löbe & AboJabel, 2022; Kiselica et al., 2024), we formulated the following hypotheses.

Non-pharmacological interventions—including cognitive stimulation therapy, occupational approaches, and exercise—have been shown to enhance quality of life (QoL) and related outcomes in early dementia ( Spector et al., 2003; Aguirre et al., 2013; Chen et al., 2022). In parallel, smart environments and ambient-assisted living (AAL) solutions promote autonomy, safety, and in-home support, reinforcing their potential to improve everyday functioning and QoL ( Rashidi & Mihailidis, 2013; Sánchez et al., 2017). Accordingly, we posit:

H1.
Implementing non-pharmacological interventions supported by smart environments and assistive technologies will significantly improve perceived QoL among older adults with early-stage dementia.

For caregivers, eHealth and technology-enabled supports have been linked to better coordination, reduced burden or isolation, and greater perceptions of safety and autonomy ( Boots et al., 2014; Mao et al., 2023; Rashidi & Mihailidis, 2013; Sánchez et al., 2017). Thus, we hypothesize:

H2.
Integrating smart-environment features (e.g., teleassistance, cognitive stimulation, motion-based technologies) will be positively associated with caregivers’ perceived autonomy, safety, and emotional well-being.

Finally, consistent with the Technology Acceptance Model (TAM)—which posits that perceived usefulness and ease of use influence technology adoption ( Davis, 1989; Holden & Karsh, 2010)—simpler, low-burden tools tend to show higher acceptance than complex systems. Evidence on socially assistive robots remains mixed (feasible and acceptable but with inconsistent QoL effects), often resulting in lower acceptability compared to simpler technologies ( Bemelmans et al., 2012; He et al., 2022; Yu et al., 2022). Therefore:

H3.
The acceptability of smart technologies among caregivers and patients will be higher for simple, low-burden tools (e.g., reminders, geolocation, video calls) than for complex devices (e.g., robotic companions).

## Methods

### Study design

We used a pragmatic, sequential exploratory mixed-methods design with two components: (1) a focused evidence synthesis on technology-supported dementia care; and (2) a cross-sectional survey of caregivers and patients to assess needs, acceptability, and current practices in a Colombian setting.

### Participants and setting

Participants were caregivers and patients in contact with a dementia care foundation in Montería (Córdoba, Colombia). Eligibility included age ≥18 years and the ability to provide informed consent (directly or via a legally authorized representative for patients).

Ethics approval and informed consent. The protocol involved an anonymous, minimal-risk questionnaire and open-ended items. In line with Colombian Ministry of Health Resolution 8430 of 1993 for minimal-risk research, no formal institutional review board approval was sought and no approval/waiver identifier is available. All participants received study information and provided informed consent before participation; consent was documented in written form (including electronic consent for remote completion). Where a participant had potentially reduced decision-making capacity, consent was obtained from a legally authorised representative and assent was sought when feasible.

### Data collection

We collected structured survey responses on demographics, care context, technology access and usage, perceived needs, and priorities for smart-environment features. Open-ended questions captured qualitative insights on barriers and facilitators.

### Temporal coverage and timestamp handling

The instrument dataset comprised 51 timestamped responses collected from January 29, 2022 to April 29, 2025 (≈ 1,187 days of coverage), with 0% missing timestamps. All times were recorded in local time (America/Bogota, UTC−05: 00) and stored in ISO 8601/RFC 3339 format; prior to analysis, timestamps were normalized to UTC and screened for gaps, duplicates, and monotonicity. See Table 7 for temporal coverage and the timestamp standard.

### Measures and variables

The survey dataset comprised 51 records and 10 variables. Data were collected using an ad hoc questionnaire originally designed and validated by Romero-Torres (2025) as part of his doctoral research on smart-environment–based care for dementia. The instrument consisted of 11 items organized into dimensions addressing demographic data, caregiving context, access to assistive technologies, perceived needs, and priorities for technological adoption.

The questionnaire’s content validity was established through expert judgment by specialists in psychology, biomedical engineering, and gerontology, ensuring conceptual clarity and cultural appropriateness. For the present study, the instrument was adapted to the Colombian context, maintaining its theoretical consistency while refining wording to improve comprehension among caregivers and patients.

Variables included a mix of categorical fields (e.g., respondent role, access to devices, perceived needs) and numeric fields (e.g., age, caregiving hours). This structure supported both quantitative analysis and qualitative synthesis, consistent with the mixed-methods design of the study.

### Data analysis

We conducted a two-strand analysis aligned with the study hypotheses:

(i) **Quantitative strand**. We computed descriptive statistics for categorical variables (counts, percentages) and numeric variables (mean, SD, and quantiles). To test H1–H3, we examined associations between smart-environment/assistive-technology use (and acceptability) and perceived outcomes (quality of life, autonomy, safety, emotional well-being):
  • **Group comparisons:** Pearson’s χ ^2^ or Fisher’s exact tests (categorical outcomes); Welch’s *t* test or Mann–Whitney *U* (two groups) and Kruskal–Wallis (≥3 groups) for ordinal/continuous outcomes, depending on normality (Shapiro–Wilk) and variance homogeneity (Levene).
  • **Association/effect size:** Cramér’s *V* (categorical), Cohen’s *d* (parametric) or Cliff’s delta (non-parametric), and rank-biserial correlations for ordinal links.
  • **Models (exploratory):** Robust logistic/ordinal regressions (Huber–White SEs) to estimate adjusted associations between technology use/acceptability and perceived outcomes, controlling for age, caregiver role, and caregiving hours.
  • **Multiple testing & precision:** Benjamini–Hochberg FDR control (q = 0.10) for families of tests; 95% CIs via non-parametric bootstrap (5,000 resamples) where applicable.
  • **Missing data:** Pairwise deletion for ≤5% missingness; otherwise, single imputation with predictive mean matching for continuous and modal imputation for categorical sensitivity checks.
(ii) **Qualitative strand.** Open-ended responses were analyzed using inductive–deductive thematic analysis, with a codebook mapped a priori to H1–H3 (e.g., low-burden tools → acceptability; safety/reminders → perceived autonomy/quality of life). Two coders conducted independent coding, reconciled discrepancies by consensus, and generated higher-order themes (credibility checks: coder agreement logs and audit trail).

**Integration.** We used a convergent narrative to triangulate quantitative signals (effects/associations) with qualitative themes (barriers, facilitators, and perceived value). Analyses were performed in Python (pandas, numpy, scipy, statsmodels).

## Results

This analysis is based on a sample of 51 records (10 variables) combining responses from caregivers and patients. Given the sample composition, inference should be cautious: the present results are descriptive and intended to contextualize hypotheses H1–H3. Below are the main sociodemographic characteristics and the distribution of knowledge and technologies reported, which will help guide qualitative interpretation and subsequent association analyses.

Descriptive characteristics are summarised in Tables 1- 3 (sex, caregiver role, and marital status) and Table 4 (knowledge domains). Preferences for smart-environment technologies are summarised in Table 5, while the age distribution is described in Table 6 and temporal coverage in Table 7. Inferential comparisons and association tests are reported in Tables 8- 14.

**Table 1.** Value counts for Sex (N = 51).

Sex	Count	Percent
**Female**	31	60.8%
**Male**	20	39.2%

**Table 2.** Value counts for Caregiver role (N = 51).

Role	Count	Percent
Family member	36	70.6%
Paid caregiver	12	23.5%
Patient	3	5.9%

**Table 3.** Value counts for Marital status (N =51).

Marital status	Count	Percent
Single	28	54.9%
Married	10	19.6%
Domestic partnership	9	17.6%
Separated	4	7.8%

**Table 4.** Value counts for knowledge domains marked about dementia (N =46).

Combination of domains	Count	Percent
Disease description, Symptoms	10	21.7%
Disease description, Symptoms, Stages	10	21.7%
Disease description, Etiology, Symptoms, Stages	8	17.4%
Disease description	4	8.7%
Symptoms	3	6.5%
Stages	3	6.5%
Etiology, Symptoms, Stages	2	4.3%
Etiology, Stages	2	4.3%
Symptoms, Stages	2	4.3%
Etiology	2	4.3%

*Note*: Combinations of: Disease description, Etiology, Symptoms, Stages.

**Table 5.** Value counts for virtual reality, augmented reality and mixed reality; Tele-assistance and Tele-surveillance; Cognitive Tele-stimulation.

Virtual Reality, Augmented Reality and Mixed Reality, Tele-assistance and Tele-surveillance, Cognitive Tele-stimulation	Count	Percent
Virtual Reality, Augmented Reality and Mixed Reality	8	21.6
Tele-assistance and Tele-surveillance	6	16.2
Tele-assistance and Tele-surveillance, Cognitive Tele-stimulation	6	16.2
Tele-assistance and Tele-surveillance, Robotic Pets	3	8.1
Virtual Reality, Augmented Reality and Mixed Reality, Tele-assistance and Tele-surveillance	3	8.1
Virtual Reality, Augmented Reality and Mixed Reality, Cognitive Tele-stimulation, Motion-based Technologies	3	8.1
Cognitive Tele-stimulation, Audio-based Technologies	2	5.4
Cognitive Tele-stimulation	2	5.4
Motion-based Technologies	2	5.4
Virtual Reality, Augmented Reality and Mixed Reality, Tele-assistance and Tele-surveillance, Cognitive Tele-stimulation, Motion-based Technologies, Audio-based Technologies, Robotic Pets	2	5.4

**Table 6.** Age summary (with normality & outliers).

Variable	n	Mean	SD	Median	IQR (Q1–Q3)	Min	Max	Shapiro–Wilk p	Outliers (IQR rule)
Age (years)	51	35.96	15.21	33	24–45	18	86	0.0011	1

*Note:* Outliers flagged using Tukey’s rule (≥ Q3 + 1.5×IQR or ≤ Q1 − 1.5×IQR). The Shapiro–Wilk test indicates non-normality ( *p* = 0.0011); use Mann–Whitney U (or other nonparametric tests) for age comparisons.

**Table 7.** Temporal coverage (Timestamp).

Field	Standardized format	Earliest timestamp	Latest timestamp	Time zone (IANA)	N records	Missing (%)	Coverage (days)
Timestamp	ISO 8601/RFC 3339 (YYYY-MM-DD THH:MM:SS ±HH:MM)	2022-01-29 11:20	2025-04-29 17:06	America/Bogota (UTC-05:00)	51	0%	1 187

*Note:* The instrument dataset contains 51 timestamped responses collected between January 2022 and April 2025. All timestamps were recorded in local time (America/Bogota, UTC-05:00) and stored in ISO 8601/RFC 3339 format. No daylight-saving adjustments apply. Data were validated for monotonicity and completeness prior to analysis.

**Table 8.** Mann–Whitney U tests for Age (years).

Comparison	Group A (n)	Group B (n)	Median A (IQR)	Median B (IQR)	U	p (two-sided)	Rank-biserial r
Used any technology (Yes vs No)	46	6	33 (23–44)	36 (28–45)	107	0.3898	0.221
Knows any technology (Yes vs No)	49	3	34 (24–44)	31 (29–58)	58	0.5557	0.211
Sex (Female vs Male)	32	20	32 (25–41)	33 (19–52)	337	0.7561	−0.053
Role (Caregiver vs Family)	13	36	38 (34–47)	26 (22–37)	356	0.0059	−0.521

*Note:* Mann–Whitney U tests compare Age distributions between the two groups per row. Rank-biserial correlation r = 1−2UnAnBr = 1 - \frac{2U}{n_An_B}r = 1−nAnB2U is provided as an effect size (|r|≈ 0.1 small, 0.3 medium, 0.5 large). Rows with very small group sizes (e.g., n = 3 or n = 6) should be interpreted cautiously.

**Table 9.** Mann-Whitney U test results for age medians.

Comparison	Group A (n)	Group B (n)	Median A (IQR)	Median B (IQR)	U	p (two-sided)	Rank-biserial r
Role (Patient vs Family) [Age]	3	36	60 (60–60)	26 (22–37)	6	**0.017**	−0.667
Within Used = Yes: Knows (Yes vs No) [Age]	27	19	34 (26–45)	32 (22–44)	236	0.491	0.111
Within Knows = Yes: Used (Yes vs No) [Age]	27	22	34 (26–45)	33 (24–43)	292	0.818	0.030

*Note:* Mann–Whitney U compares age distributions between the listed groups. Rank-biserial r is an effect size (≈0.1 small, 0.3 medium, 0.5 large). Rows with very small groups (e.g., patients, n = 3) should be interpreted cautiously *.*

**Table 10.** Mann–Whitney U tests for age across roles, knowledge, and use (two-sided).

Comparison	Group A (n)	Group B (n)	Median A (IQR)	Median B (IQR)	U	p (two-sided)	Rank-biserial r
Used any technology (Yes vs No) [Age]	46	6	33 (23–44)	36 (28–45)	107	0.3898	0.221
Knows any technology (Yes vs No) [Age]	49	3	34 (24–44)	31 (29–58)	58	0.5557	0.211
Sex (Female vs Male) [Age]	32	20	32 (25–41)	33 (19–52)	337	0.7561	−0.053
Role (Caregiver vs Family) [Age]	13	36	38 (34–47)	26 (22–37)	356	**0.0059**	−0.521
Role (Patient vs Family) [Age]	3	36	60 (60–60)	26 (22–37)	6	**0.0171**	−0.667
Role (Caregiver vs Patient) [Age]	13	3	38 (34–47)	60 (60–60)	4	**0.0362**	0.513
Within Used = Yes: Knows (Yes vs No) [Age]	27	19	34 (26–45)	32 (22–44)	236	0.4914	0.111
Within Knows = Yes: Used (Yes vs No) [Age]	27	22	34 (26–45)	33 (24–43)	292	0.8178	0.030
Family only: Used (Yes vs No) [Age]	33	3	26 (22–37)	31 (29–58)	41	0.2548	0.212
Caregiver only: Used (Yes vs No) [Age]	12	1	38 (34–47)	29 (—)	6	0.5263	0.167
Family only: Knows (Yes vs No) [Age]	33	3	26 (22–37)	31 (29–58)	41	0.2548	0.212
Caregiver only: Knows (Yes vs No) [Age]	12	1	38 (34–47)	29 (—)	6	0.5263	0.167

*Note:* Medians and IQRs are in years. Rank-biserial r is an effect-size measure for Mann–Whitney (|r|≈ 0.1 small, 0.3 medium, 0.5 large). Results involving very small groups (e.g., n = 1–3 for patients or “No” cells) should be interpreted cautiously. Significant differences at α = .05 are bolded. The age distribution is skewed; nonparametric tests were used by design *.*

**Table 11.** Categorical associations (χ ^2^/Fisher), with Cramér’s V and BH–FDR.

Comparison	Method	df	Statistic	p	Cramér’s V	q (BH, 0.10)
Used any technology × Role	Pearson χ ^2^	2	12.793	0.0019	0.503	0.0075
Knows any technology × Role	Pearson χ ^2^	2	3.005	0.2220	0.243	0.4440
Used any technology × Sex	Fisher exact (2×2)	—	—	0.4294	0.140	0.5726
Knows any technology × Sex	Fisher exact (2×2)	—	—	0.6120	0.124	0.6120

*Note:* Pearson χ ^2^ without continuity correction; Fisher’s exact test for 2×2 tables. Cramér’s V is reported as the effect size (≈0.1 small, 0.3 medium, 0.5 large). Multiple-testing adjustment uses Benjamini–Hochberg (target q = 0.10). The Use × Role association is statistically significant with a large effect (V ≈ 0.50).

**Table 12.** Age across roles (Kruskal–Wallis) and pairwise Mann–Whitney with effect sizes.

Group	n	Median	IQR (Q1–Q3)	Kruskal–Wallis H	p
Family	36	26	22–37	**11.724**	**0.0028**
Caregiver	13	38	34–47		
Patient	3	60	60–60		

**Table 13.** Pairwise tests (two-sided).

Pair	nA	nB	Median A (IQR)	Median B (IQR)	U	p	Rank-biserial r	Cliff’s δ	q (BH, 0.10)
Caregiver vs Family	13	36	38 (34–47)	26 (22–37)	356	**0.0059**	**−0.521**	−0.518	**0.0089**
Patient vs Family	3	36	60 (60–60)	26 (22–37)	6	**0.0171**	**−0.667**	−0.667	**0.0257**
Caregiver vs Patient	13	3	38 (34–47)	60 (60–60)	4	**0.0362**	**0.513**		

*Notes **.*** Nonparametric tests were used due to age skewness. Effect sizes are reported as rank-biserial r and Cliff’s δ (|r|≈ 0.1 small, 0.3 medium, 0.5 large). Cells with very small n (e.g., the patient group) warrant cautious interpretation; findings should be corroborated with sensitivity analyses where feasible. Benjamini–Hochberg FDR (q = 0.10) was applied across the three contrasts.

**Table 14.** Bootstrap 95% CIs for rank-biserial r (key contrasts, B =1,000).

Contrast	nA	nB	r (rank-biserial)	95% CI (percentile)
Used: Yes vs No (Age)	46	6	0.221	[−0.202, 0.561]
Knows: Yes vs No (Age)	49	3	0.211	[−0.399, 0.625]
Role: Caregiver vs Family	13	36	−0.521	[−0.726, −0.205]

The sample is predominantly female (31/51; 60.8%). This female predominance aligns with global caregiving literature, where women assume most family caregiving responsibilities. From the perspective of our hypotheses, this overrepresentation of women may influence perceptions of acceptability and technological needs (H2 and H3): for instance, training and adoption strategies should account for gender roles and responsibilities to improve adherence to non-pharmacological support tools.

The majority (70.6%) are family caregivers, 23.5% are paid caregivers, and 5.9% are patients who responded on their own behalf. This distribution supports the need to focus recommendations and guidelines on domestic/family settings rather than only institutional environments. It also aligns with H2: perceived safety and autonomy are often strongly mediated by family caregivers, so any associations between technologies (e.g., telecare, sensors) and perceived outcomes should control for caregiver type in later analyses.

More than half of respondents (54.9%) listed themselves as single, while 19.6% are married. Because the sample mixes caregivers and patients, this composition suggests heterogeneity in living situations (living alone vs. cohabitation), which can affect technological priorities (e.g., the need for geolocation or fall detection may be greater among those who live alone). Recommendation: cross-tabulate marital status with caregiver role to identify subgroups with different needs.

Among the 46 valid responses on knowledge domains, the combinations “Disease description + Symptoms” and “Disease description + Symptoms + Stages” are the most frequent (each 21.7%). This indicates a knowledge profile focused on symptom recognition rather than etiology or full clinical staging. Practical implication: the intervention materials should include short, stage-oriented education modules to close knowledge gaps and facilitate appropriate use of tools (supporting H1 and H2 — quality of life and autonomy improvements require caregivers to understand stage-specific expectations).

The technologies cited show a mix of interests **:** Virtual/Augmented/Mixed Reality is the most frequently mentioned single category (8 responses; 21.6%), followed by Tele-assistance and Tele-surveillance (6; 16.2%) and combinations that include cognitive tele-stimulation (6; 16.2%). Interpret cautiously: these counts reflect awareness/mention of technologies, not necessarily acceptance or feasibility. While H3 predicts greater acceptability for low-effort tools (reminders, video calls), the notable presence of extended-reality solutions suggests curiosity or expectation about advanced solutions. Therefore, analyze actual acceptability (a specific questionnaire item) by technology and cross it with age, caregiver role, and knowledge level before drawing conclusions about preferences.

Table 6 shows that the sample’s age distribution is right-skewed (Shapiro–Wilk p = 0.0011), with a single outlier (86 years) by Tukey’s rule. This justifies nonparametric comparisons in subsequent analyses. Importantly, age does not appear to drive technology familiarity or use: Mann–Whitney tests found no significant age differences between those who used any technology vs. not, or those who knew about technologies vs. not (all p > .38; small rank-biserial |r| ≈ 0.21). Thus, acceptance/engagement with simple, low-burden tools is not merely a function of being younger, lending support to H3 (higher acceptability for simple tools regardless of age).

By contrast, role groups differ in age—as expected in real-world caregiving contexts. Caregivers are older than family members (median 38 vs. 26 years; *p* = 0.0059; large | *r*| ≈ 0.52), and patients are oldest (median 60 vs. 26 years vs. family; *p* = 0.017). These gradients align with H2, where perceived autonomy/safety benefits accrue in dyads that include older caregivers and patients who are most exposed to smart-environment risks and supports. Crucially, because age distributions are skewed and differ by role, we report nonparametric tests throughout and recommend sensitivity checks (excluding the single age outlier) to confirm that the observed patterns—and the evidence supporting H2–H3—are robust to age-related confounding.

The temporal structure of the dataset was evaluated to ensure completeness and reproducibility. Table 7 details the timestamp standard, earliest and latest records, and the time-zone specification adopted during preprocessing.

Across “Used any technology” and “Knows any technology,” age did not differ significantly (both p > .38; small |r| ≈ 0.21). Together with Table 6, this suggests that basic familiarity/uptake of smart tools is not primarily age-driven in this sample—supporting H3, which posits higher acceptability for simple, low-burden solutions regardless of age. There is also no age difference by sex (p = .756; |r| ≈ .05), indicating sex is unlikely to confound age-related interpretations.

By contrast, caregivers are older than family members (median 38 vs. 26 years; *p* = .0059; | *r*| ≈ .52—large). This role-related age gradient is expected and relevant for H2 (perceived autonomy/safety): analyses of role effects should therefore use nonparametric methods and either (i) stratify by role or (ii) adjust for age (e.g., median split sensitivity, rank-based ANCOVA, or permutation tests) to ensure that any benefit attributed to smart-environment features is not merely an artifact of older caregiver age.

In the comparison between patients and family members, patients were significantly older (Mann–Whitney *p* = .017; large rank-biserial effect size | *r*| ≈ .67). This role–age gradient is expected in dementia research and has implications for H2: analyses examining associations between smart-environment features and perceived autonomy/safety should account for role and age—via stratification or statistical adjustment—to mitigate confounding.

Stratified contrasts (technology knowledge within the subgroup that reported use, and technology use within the subgroup that reported knowledge) were non-significant (all *p* > .49; very small| *r*|). Considered alongside Tables 7 and 9, these results indicate that age is unlikely to be the primary determinant of technology familiarity or uptake among participants already engaged with technology, which supports H3: simpler, low-burden tools tend to be acceptable across age groups.

Table 10 indicates that age does not differ meaningfully between participants who report using any technology versus not, nor between those who know about such technologies versus not (all p ≥ .39; small rank-biserial |r| ≈ .21). The same holds across sex (p = .76). Taken together, these findings suggest that age is not the primary driver of basic technology familiarity or uptake in this sample—bolstering H3, which posits higher acceptability for simple, low-burden tools across age groups.

By contrast, role-based comparisons reveal clear age gradients: caregivers are older than family members (median 38 vs. 26 years; *p* = .0059; large | *r*| ≈ .52), and patients are oldest relative to family (median 60 vs. 26 years; *p* = .017; large | *r*| ≈ .67). Caregivers also differ from patients ( *p* = .036; | *r*| ≈ .51). These patterns are expected in dementia care and are highly pertinent to H2 (perceived autonomy/safety). Any observed advantages of smart-environment features for caregivers and patients should therefore control for role and age (e.g., stratification by role, covariate adjustment in regression, or rank-based ANCOVA/permutation methods) to mitigate confounding.

Stratified tests (knowledge within users; use within those with knowledge; and role-specific contrasts within family vs. caregiver subgroups) are uniformly non-significant (all *p* ≥ .25; very small | *r*|), reinforcing the inference that, among individuals already engaged or informed, age contributes little to differences in technology engagement—again consistent with H3.

Given the skewed age distribution and some very small cells (e.g., patients *n* = 3; “No” groups), it is important to: (i) emphasize exact *p* values and effect sizes (rank-biserial *r*), (ii) flag small- *n* comparisons for cautious interpretation, and (iii) conduct sensitivity analyses (e.g., excluding the single extreme age or using robust rank-based methods) to confirm that the associations underpinning H2–H3 are not artifacts of age distribution or sparse strata.

In line with H2, the large and statistically significant association between technology use and participant role indicates that engagement with smart-environment solutions is role-dependent. That is, patients, caregivers, and family members exhibit distinct patterns of uptake, which is consistent with the expectation that the caregiving context shapes both needs and opportunities to deploy teleassistance, motion-based sensors, and cognitively oriented tools. Methodologically, this justifies role-aware analyses (e.g., stratification or covariate adjustment) when estimating links between smart-environment features and perceived autonomy and safety.

Regarding H3, the absence of significant differences by sex in both technology use and knowledge suggests that gender is not a primary determinant of familiarity or uptake. This pattern accords with technology-acceptance accounts emphasizing perceived usefulness and ease of use over basic demographics as drivers of adoption. Together with the modest, non-significant “Know × Role” association, these results are compatible with the notion that simple, low-burden tools—such as reminders, geolocation, or video calls—can achieve broad acceptability across user groups once exposure occurs.

Finally, with respect to H1, the observed role-related differences in use imply that subsequent analyses linking technology exposure to quality of life (QoL) must adjust for role (and age) to mitigate confounding. Such adjustment will strengthen the interpretability of any observed QoL benefits attributable to non-pharmacological, technology-supported care.

The age distribution differs significantly across roles (Kruskal–Wallis H = 11.724, p = 0.0028), with family members being younger (median = 26, IQR = 22–37), caregivers older (median = 38, IQR = 34–47), and patients oldest (median = 60, IQR = 60–60). This graded pattern substantiates the premise in H2 that the caregiving context is intrinsically shaped by age-related needs and responsibilities: older participants—particularly patients and, to a lesser extent, caregivers—are more likely to interact with smart-environment functions aimed at safety, monitoring, and support, whereas younger family members may engage differently (e.g., coordination, remote assistance). Analytically, the strong role–age structure motivates role- and age-adjusted models (or stratification) when linking smart-environment features to perceived autonomy and safety, thereby reducing confounding and improving internal validity of the H2 tests.

For H1, the same role–age gradient implies that any observed improvements in quality of life (QoL) associated with non-pharmacological, technology-supported care could be partially attributable to age and role composition. Accordingly, subsequent QoL analyses should adjust for role and age (and, where possible, caregiving intensity) to isolate the incremental contribution of smart environments to QoL outcomes.

With respect to H3, the between-role age differences indicate that acceptance and uptake must be interpreted in light of age—yet they do not, by themselves, contradict the hypothesis that simple, low-burden tools will be broadly acceptable. Instead, the findings underscore the need to disentangle age/role effects from perceived usefulness/ease-of-use in acceptance analyses (e.g., by controlling for age and role when estimating associations between tool simplicity and acceptability). Finally, given the small number of patients (n = 3), all inferences involving the patient group should be interpreted cautiously and, where feasible, corroborated via sensitivity analyses (e.g., robust nonparametric tests, influence checks, or resampling).

Pairwise comparisons reveal a clear, role-dependent age gradient. Caregivers are significantly older than family members (U = 356, p = 0.0059, q = 0.0089), with a medium-to-large effect (r = −0.521; Cliff’s δ = −0.518). Patients are markedly older than family members as well (U = 6, p = 0.0171, q = 0.0257; r = −0.667; δ = −0.667). In turn, patients are older than caregivers (U = 4, p = 0.0362, q = 0.0362; r = 0.513; δ = 0.513). This ordered progression (Family < Caregiver < Patient) is consistent across tests and effect-size metrics, indicating robust and directionally coherent differences.

Substantively, this pattern is aligned with H2: the caregiving context—often taken on by older individuals and centred on the needs of even older patients—shapes both the demand for, and interaction with, smart-environment functions aimed at autonomy and safety (e.g., teleassistance, monitoring, and cognitively oriented supports). Methodologically, these findings motivate role- and age-aware analyses (via covariate adjustment or stratification) when estimating links between smart-environment features and perceived outcomes, thereby limiting confounding attributable to the role–age structure.

The observed age–role structure has implications beyond H2. For H1, any improvements in quality of life (QoL) associated with non-pharmacological, technology-supported care must be interpreted within this demographic context, as part of the observed QoL variance may arise from differences in role and age composition rather than from the intervention itself. For H3, acceptance and uptake patterns should be analyzed with explicit control for age and role to separate intrinsic acceptance drivers—such as perceived usefulness and simplicity—from structural determinants (role, age, caregiving intensity). Together, these findings validate the theoretical model guiding this study: age and role jointly shape engagement with smart environments, influence perceived autonomy and QoL, and condition technology acceptance across caregiving actors.

Bootstrap estimates (B = 1,000; percentile confidence intervals) reinforce the role–age structure while tempering inferences about age as a driver of basic exposure. For Use: Yes vs No, the rank-biserial correlation is modest (r = 0.221) and its 95% CI includes zero [−0.202, 0.561], providing no precise evidence that age alone differentiates users from non-users. A similar pattern holds for Knows: Yes vs No (r = 0.211; 95% CI [−0.399, 0.625]), again suggesting that age is not the primary determinant of technology familiarity once some exposure exists.

By contrast, Caregiver vs Family yields a medium-to-large effect (r = −0.521) with a 95% CI that does not cross zero [−0.726, −0.205], confirming a robust age difference between these roles. This finding is consistent with the omnibus and pairwise role comparisons and underscores a stable, ordered role–age gradient in the sample.

Implications for the hypotheses. In relation to H2, the robustness of the caregiver–family age difference supports the view that the caregiving context—and its age structure—conditions engagement with smart-environment features pertinent to autonomy and safety. Accordingly, subsequent analyses linking features to perceived outcomes should adjust for role and age (or stratify) to reduce confounding. For H3, the wide, zero-spanning CIs for Use and Know by age align with the premise that acceptance is governed more by perceived usefulness and ease of use than by age per se, consistent with higher acceptability of simple, low-burden tools across age groups once exposure occurs. With respect to H1, because role (and thus age) predicts patterns of engagement, any association between technology-supported, non-pharmacological care and quality of life should be modeled with role/age controls to isolate the incremental contribution of smart environments.

In summary, the sample is predominantly female (≈32 female vs. 20 male) and primarily family-based in caregiving roles (Family = 36; Caregivers = 13; Patients = 3). Technology familiarity and use are widespread (Knows = 49/52; Used = 46/52), but use varies markedly by role (Use × Role: χ ^2^(2)=12.79, p=0.0019, Cramér’s V=0.50, large). The age structure is strongly role-graded (Kruskal–Wallis H=11.72, p=0.0028): Family members are younger (median = 26, IQR = 22–37), Caregivers older (median = 38, IQR = 34–47), and Patients oldest (median = 60). Pairwise tests confirm large effects (e.g., Patients > Family, p=0.017; Caregivers > Family, p=0.0059). By contrast, sex is not associated with either knowledge or use (Fisher’s p≥0.43), and bootstrap intervals for age differences by “Use”/“Know” span zero, suggesting that age per se is not the primary driver of basic exposure once individuals are engaged.

Regarding knowledge content, responses concentrate on disease description and symptom recognition, while the technology items reveal interest in both advanced solutions (e.g., VR/AR) and practical tools (e.g., tele-assistance, monitoring). These features of the sample motivate the inferential strategy that follows: analyses will examine associations between role, age, dementia knowledge, and technology acceptability/use, with role- and age-adjustment (or stratification) to address the pronounced role–age gradient. This approach directly targets H1–H3: isolating the contribution of smart, non-pharmacological supports to quality of life (H1); assessing links between smart-environment features and perceived autonomy/safety among caregivers (H2); and evaluating whether simple, low-burden tools exhibit broad acceptability beyond basic demographics (H3).

## Discussion

Our findings indicate a clear preference among caregivers and patients for simple, low-burden technologies that address everyday needs—timely reminders, safety and monitoring, and social connection—over more complex devices that may impose usability burdens. This pattern is consistent with the broader literature foregrounding acceptability, personalization, and caregiver support in technology-enabled dementia care. At the same time, effective implementation in resource-constrained settings requires attention to connectivity, basic digital literacy, and cultural adaptation to ensure sustainable uptake and equitable access.

Empirically, we observed a large Use × Role association (Cramér’s V ≈ 0.50) and a strong role–age gradient (Family < Caregiver < Patient) with significant pairwise differences and medium-to-large effect sizes. By contrast, sex was not associated with technology knowledge or use, and bootstrap confidence intervals for age differences by “Use” and “Know” overlapped zero. Taken together, these results suggest that who the participant is in the care network (role)—and the age distribution embedded in those roles—better explains patterns of exposure and use than age or sex alone. This aligns with H2, which posits that the caregiving context shapes engagement with smart-environment features linked to perceived autonomy and safety. It also supports H3: once individuals are exposed, perceived usefulness and ease of use—rather than demographics—appear to drive acceptance, which helps explain the cross-role appeal of low-friction tools (e.g., teleassistance, reminders, basic geolocation, video calls).

The theoretical synthesis from Romero-Torres (2025) supports these empirical trends. His mixed-methods work reports that non-pharmacological interventions delivered through smart environments enhance emotional stability, autonomy, and functional capacity among older adults with early-stage dementia. Integrations such as teleassistance systems, tablet-based cognitive stimulation, and wearable sensors improved daily routines and strengthened patient–caregiver emotional connection, echoing our pattern of preference for tools that are immediately actionable in the home context. This is consonant with Löbe and AboJabel (2022), who show that intelligent assistive technologies promote empowerment and self-efficacy, mechanisms that plausibly mediate quality-of-life (QoL) gains anticipated in H1.

Equally important is the psychosocial dimension. As De Oliveira (2023) argues, ethical health care requires clinical empathy and patient-centered design. These principles are especially salient in real-world Colombian contexts, characterized by limited resources and heterogeneous cultural perceptions of aging and dementia. In this light, the methodological guide developed here functions as both a technological and pedagogical instrument—supporting caregivers to select and use tools that fit their competencies and their relatives’ needs, while respecting local norms and expectations.

Beyond the clinical and human factors, the legal and institutional framework in Colombia (Constitution of 1991; Laws 1616/2013, 2055/2020, and 2460/2025) recognizes the right to dignified aging and mental health care. Against this backdrop, the integration of assistive technologies is not only a scientific and service-delivery innovation but also a contribution to public policy and social inclusion. As Val and Cardoso (2021) emphasize, embedding digital health competencies into the training of caregivers and health professionals is crucial for long-term sustainability, a finding that resonates with our implementation-focused recommendations.

Methodologically, our analytic choices addressed the empirical structure of the data: non-parametric tests (Shapiro–Wilk non-normality for age), effect sizes (Cramér’s V, rank-biserial r, Cliff’s δ), bootstrap CIs for robustness, and Benjamini–Hochberg FDR to control multiplicity. Given the strong role–age gradient, we highlight the importance of role- and age-adjusted models (or stratification) when estimating associations between smart-environment features and outcomes pertinent to H1 (QoL) and H2 (autonomy/safety), thereby improving internal validity. Our results also point toward H3-consistent mechanisms (usefulness and ease-of-use) that can be explicitly modeled in future work (e.g., mediation analyses or technology-acceptance constructs) to clarify pathways from design features to acceptability and sustained use.

Limitations include the small number of patients (n = 3) and unbalanced group sizes, the reliance on self-report for knowledge/use, and the cross-sectional design, which limits causal inference. We mitigated these constraints via robust statistics and caution in interpretation, but future research should pursue larger, balanced samples, include objective usage data, and consider longitudinal or pragmatic trial designs to estimate within-person changes in QoL and caregiver outcomes.

Overall, this study advances theoretical understanding by linking technological empowerment, non-pharmacological therapy, and cognitive rehabilitation under a paradigm of pragmatic care innovation. The proposed methodological guide operationalizes this linkage in a replicable format suitable for institutions across Latin America. Consistent with our theoretical propositions and prior empirical literature, the evidence supports our initial hypothesis: a methodological guide grounded in smart environments and assistive technologies can feasibly improve autonomy, emotional well-being, and overall quality of life for older adults with early-stage dementia in Colombia. These results underscore the need to consolidate an ethical, humanized, and sustainable approach to geriatric innovation—one that harmonizes empirical evidence, cultural context, and technological adaptability—while remaining aligned with national policy commitments and the everyday realities of caregivers and families.

### Limitations

This study has several limitations that should be considered when interpreting the findings. First, the cross-sectional and self-reported nature of the data constrains causal inference and temporal generalization. Although the sequential exploratory design integrated qualitative and quantitative components, the sampling frame was geographically restricted to Montería (Córdoba, Colombia), which may limit external validity across other Latin American contexts. Larger, multi-site samples are needed to assess reproducibility and generalizability.

Second, despite adherence to good-practice reporting principles (e.g., STROBE/COREQ/PRISMA), mixed-methods work in pragmatic settings entails interpretive challenges. As Romero-Torres (2025) notes, human–technology interaction in dementia care is complex and not fully captured by quantitative indicators alone. Contextual variables—family dynamics, cultural views of aging, and digital literacy—likely influenced both responses and outcomes, and were not comprehensively measured here.

Third, the empirical structure of our dataset imposes analytic constraints. Age was non-normally distributed (Shapiro–Wilk p ≈ 0.001), prompting nonparametric tests by design; nevertheless, very small cell sizes—most notably the patient group (n = 3) and some “no/none” categories—reduce power and precision, as reflected in wide bootstrap confidence intervals for several contrasts. We mitigated multiplicity via Benjamini–Hochberg FDR (q = 0.10) and reported effect sizes, but the combination of multiple tests and small n increases the risk of both Type I and Type II error. In addition, the strong role–age gradient and the large Use × Role association raise the possibility of residual confounding by role-linked factors (e.g., caregiving intensity) not fully captured in our measures.

Fourth, technological access and infrastructure disparities remain a structural barrier in Colombia. Limited broadband coverage, device affordability, and gaps in digital training can constrain scalability of smart-environment interventions—an issue also highlighted by Val and Cardoso (2021). While our instruments were adapted to enhance local comprehension, psychometric validation is pending, which may affect measurement precision and comparability across settings.

Fifth, the absence of longitudinal follow-up precludes assessment of sustained behavioral, cognitive, or caregiver outcomes. As Löbe and AboJabel (2022) emphasize, empowerment and independence require repeated measures to determine whether benefits from intelligent assistive technologies persist beyond short-term adoption. Similarly, emotional and psychosocial dimensions—central to ethical caregiving per De Oliveira (2023)—were only partially assessed here and warrant deeper evaluation to understand long-term impact on caregiver well-being.

Finally, the study did not directly compare pharmacological versus non-pharmacological approaches. Our focus on non-pharmacological therapy was intentional to foreground social, cognitive, and environmental determinants of quality of life; nonetheless, combined or comparative designs could enrich future work by clarifying complementarities and trade-offs.

Addressing these limitations—through larger, more diverse samples, validated instruments, objective usage metrics, and longitudinal or pragmatic evaluations (e.g., implementation outcomes, cost-effectiveness)—will strengthen the empirical basis for the methodological guide and enhance its scalability as a model for inclusive, technology-assisted dementia care in Latin America.

### Implications and next steps

The integration of assistive technologies and smart environments for dementia care in Colombia presents immediate practical avenues and broader theoretical implications. Empirically, we documented a large Use × Role association and a graded role–age structure (Family < Caregiver < Patient), alongside null sex differences in knowledge and use and wide, zero-spanning bootstrap CIs for age when comparing users vs. non-users. Taken together, these patterns support a pragmatic model of innovation that bridges technology, human care, and psychosocial well-being while underscoring the need for role- and age-aware implementation. Building on the doctoral framework proposed by Romero-Torres (2025), the methodological guide derived here functions both as a clinical instrument and as a pedagogical scaffold to promote adoption through education, digital inclusion, and empathic practice.

### Practical implications

At the institutional level, non-pharmacological care grounded in smart environments can help structure daily routines for older adults and reduce caregiver burden, particularly for caregiver and patient subgroups that our data show are older and more directly engaged with risk-mitigating features. Consistent with Val and Cardoso (2021), technology literacy should be embedded in training for professional and family caregivers to ensure sustainable uptake. The guide provides operational matrices to match context-appropriate tools—teleassistance, motion sensing, and audio-based cognitive stimulation—to users’ capabilities and socioeconomic constraints, in line with H3 (acceptability driven by usefulness and ease of use) and our finding that sex and age, by themselves, are not primary drivers of basic exposure once engagement occurs.

Ethically, following De Oliveira (2023), interventions should foreground clinical empathy and the protection of human dignity. In Colombian contexts marked by uneven access to geriatric mental-health services, this entails locally adapted frameworks that combine technical training, social support, and digital equity (e.g., connectivity solutions, low-burden interfaces), thereby creating conditions under which H1 (quality-of-life benefits) and H2 (perceived autonomy/safety) can be realized and fairly distributed.

### Theoretical implications

The evidence strengthens the conceptual bridge between technological empowerment and non-pharmacological therapeutic models. Our results are consonant with Löbe & AboJabel (2022) on empowerment/self-efficacy and with the CARES-style framing in Kiselica et al. (2024), indicating that intelligent assistive technologies can enhance autonomy and emotional stability—mechanisms through which smart environments can improve quality of life (H1). The methodological design also contributes to debates on mixed-methods and pragmatic paradigms ( Romero-Torres, 2025): triangulating quantitative indicators with qualitative narratives produced a more holistic account of user needs and showed that co-design with caregivers is essential for long-term adherence (speaking directly to H3’s emphasis on low-burden, acceptable tools).

### Policy and social implications

Colombian statutes on mental health and aging (Law 1616/2013; Law 2055/2020; Law 2460/2025) provide an enabling policy environment to scale smart-environment interventions nationally. Aligning these tools with public-health priorities can strengthen community-based care networks and foster collaborative governance among universities, health institutions, and local governments. Given regional commonalities—rapid population aging alongside digital inequality—the model offers a replicable pathway for Latin America, contingent on attention to infrastructure, affordability, and capacity building.

### Future research directions

Future studies should evaluate the longitudinal effects of assistive technologies on cognitive trajectories, caregiver resilience, and cost-effectiveness. It is recommended to:

1. **Role- and age-adjusted evaluations:** Future analyses and pilots should adjust or stratify by role and age (as indicated by our Use×Role and role–age results) to obtain unbiased estimates of effects on QoL (H1) and perceived autonomy/safety (H2).
2. **Acceptance pathways (H3):** Incorporate explicit technology-acceptance constructs (perceived usefulness, ease of use) and usability testing to quantify how “simple, low-burden” designs drive uptake across roles.
3. **Implementation and equity:** Pair the guide with training curricula, low-bandwidth options, and device-access programs, addressing the infrastructure constraints flagged in practice and by Val & Cardoso (2021).
4. **Longitudinal/pragmatic trials:** Move beyond cross-sectional observation to longitudinal or pragmatic evaluations, capturing sustainability of empowerment and independence outcomes (per Löbe & AboJabel, 2022) and deeper psychosocial impacts (per De Oliveira, 2023).
5. **Measurement development:** Advance psychometric validation of locally adapted instruments to strengthen comparability and precision across settings.

In sum, the Colombian experience reported here connects theory, policy, and practice: smart-environment interventions—delivered through a methodological guide—are positioned to enhance autonomy, emotional well-being, and quality of life for older adults with early-stage dementia, while providing a scalable, ethical, and culturally grounded blueprint for the region.

By addressing these avenues, researchers can strengthen the empirical validation of the guide and contribute to a regional theory of technological humanism in dementia care. Ultimately, this approach aligns with the central hypothesis of this work: that integrating smart environments and assistive technologies into non-pharmacological therapy constitutes a feasible, ethical, and effective pathway toward dignified aging and improved quality of life in Latin America.

## Ethical considerations

**Ethics approval**. The study involved an anonymous, minimal-risk questionnaire with optional open-ended items. Direct personal identifiers were not collected, and responses were handled in de-identified form. In accordance with Colombian Ministry of Health Resolution 8430 of 1993 (minimal-risk research), formal review by an institutional review board/ethics committee was not required for this protocol; therefore no ethics approval or waiver reference number is available.

**Informed consent**. All participants were provided with study information and gave informed consent prior to participation. Consent was documented in written form (including electronic consent for remote completion). Where a participant had potentially reduced decision-making capacity, consent was obtained from a legally authorised representative and assent was sought where feasible.

**Confidentiality and participant wellbeing**. Data were stored securely and analysed in aggregate. Participants could decline to answer any question and could stop participation at any time. The underlying dataset shared for reproducibility is de-identified and prepared to minimise re-identification risk (see the Data Availability statement).

## Data Availability

Underlying data. Underlying data required to reproduce all results reported in this article (including the de-identified questionnaire responses, a data dictionary/codebook, and the analysis-ready tables used to compute descriptive and inferential statistics) are available from an open repository. Repository link: https://doi.org/10.5281/zenodo.18342190 ( Romero-Torres et al., 2025). License: CC-BY 4.0 (or CC0). To support full reproducibility, the repository will include: (i) the values underlying summary statistics (means/medians, dispersion measures, confidence intervals), (ii) the values used to build any graphs/figures, and (iii) variable descriptions (e.g., age, sex, caregiver role) in the codebook. The dataset is not embargoed and does not require a login. If any portion of the dataset cannot be shared due to privacy concerns, the restriction and an access pathway (without compromising participant confidentiality) will be described on the repository landing page and reflected in this statement. Materials supporting the study are available from the same repository as the underlying data: https://doi.org/10.5281/zenodo.18342190 ( Romero-Torres et al., 2025). Extended data include: the questionnaire (all language versions), participant information sheet, consent documentation template, codebook/coding framework for open-ended responses, reporting checklists (STROBE, COREQ), and the PRISMA flow diagram and checklist used to report the focused evidence synthesis component. Source code available from: https://github.com/eduardoph1/dementia-smart-environments Archived software available from: https://doi.org/10.5281/zenodo.18350858 License: MIT License This study adheres to recognised reporting standards to support transparency and reproducibility. The quantitative survey component is reported in line with the Strengthening the Reporting of Observational Studies in Epidemiology (STROBE) guidance. The qualitative component, based on open-ended questionnaire responses, is reported using items from the Consolidated Criteria for Reporting Qualitative Research (COREQ) where applicable to written responses. The evidence synthesis used to inform instrument development and feature selection was a focused (non-systematic) synthesis. We used the Preferred Reporting Items for Systematic Reviews and Meta-Analyses (PRISMA) 2020 checklist items to transparently report the search, screening, and inclusion process for that synthesis component; this does not imply that the overall study is a systematic review. Completed checklists and the PRISMA flow diagram are provided as extended data.
