# Supplementary material for: Non‑pharmacological care for early-stage dementia through smart environments in Colombia: a mixed‑methods study and methodological guide for caregivers and patients
Source: F1000Res. 2026 Mar 25;15:433. [Version 1] doi: 10.12688/f1000research.177177.1 (PMC13070209; doi:10.12688/f1000research.177177.1)
Supplement: Peer review history

# Reviewer report: Reviewer response for version 1

**DOI:** 10.5256/f1000research.195356.r472307

**Published:** 2026-04-11

**Authors:** Jhony Alejandro Diaz-Vallejo

**Affiliations:** 1 University of Caldas, Manizales, Colombia

In general, the manuscript is methodologically thoughtful and contextually grounded. This review concentrates on precision, reproducibility, analytical depth, and translational scalability, rather than on interpretative caution or conceptual framing.

 1. I would encourage the authors to improve clarity in the operationalization of key constructs, particularly those central to the study’s analytical framework. While constructs such as “acceptability,” “use,” “knowledge,” and “perceived needs” are frequently used, their operational definitions remain somewhat implicit. This may limit reproducibility and comparability across studies. A concise clarification within the Methods section would be beneficial.

 2. The manuscript would benefit from a clearer articulation of the integration strategy in the mixed-methods design. Although the study is described as “sequential exploratory,” the integration phase is presented rather briefly. Strengthening this component would enhance methodological transparency and align more closely with best practices in mixed-methods research.

 3. I recommend refining the presentation of results to improve readability and analytical hierarchy. Currently, the Results section is rich but occasionally dense, with multiple statistical outputs presented in sequence. Introducing clearer structuring (possibly through short subheadings aligned with H1-H3 or thematic domains) would facilitate reader navigation and strengthen interpretability.

 4. The manuscript would benefit from a more explicit distinction between “technology awareness” and “technology preference”. In several passages (e.g., where virtual/augmented reality appears frequently mentioned), there is a risk of conflating familiarity with desirability or feasibility. This distinction is analytically important, particularly for implementation.

 5. I suggest strengthening the external validity discussion through a more explicit positioning of the Colombian context within Latin America and other LMICs. While the manuscript references regional relevance, it could benefit from a more analytical comparison.

 6. The manuscript would gain depth by incorporating a brief reflection on implementation barriers beyond individual-level factors, particularly at meso- and macro-levels. While digital literacy and access are mentioned, system-level constraints (e.g., reimbursement models, interoperability, institutional adoption) are not fully explored.

 7. There is an opportunity to improve figure or visual synthesis quality, which would significantly enhance the manuscript’s impact. Given that the study produces a “methodological guide,” a schematic or conceptual model summarizing decision pathways for technology selection would be highly valuable. I would encourage the authors to include a figure with the following structure:

 -Input: Patient/caregiver profile (age, role, needs)

 -Decision layer: Priority domains (safety, reminders, communication)

 -Output: Recommended technology categories (low vs high complexity)

 -Modifiers: Access, literacy, infrastructure.

 Finally, I recommend a minor but important refinement in the Data Availability and reproducibility section. While the dataset and code are commendably shared, it would be helpful to explicitly state whether the analytical workflow is fully reproducible end-to-end.

Is the work clearly and accurately presented and does it cite the current literature?

Yes

If applicable, is the statistical analysis and its interpretation appropriate?

Partly

Are all the source data underlying the results available to ensure full reproducibility?

Partly

Is the study design appropriate and is the work technically sound?

Yes

Are the conclusions drawn adequately supported by the results?

Yes

Are sufficient details of methods and analysis provided to allow replication by others?

Yes

Reviewer Expertise:

Biostatistical, Evidence-Based Medicine, Biomedical Sciences, Clinical Medicine, Neuroanatomy

I confirm that I have read this submission and believe that I have an appropriate level of expertise to confirm that it is of an acceptable scientific standard.

# Reviewer report: Reviewer response for version 1

**DOI:** 10.5256/f1000research.195356.r474093

**Published:** 2026-04-10

**Authors:** Kevin Fernando Montoya-Quintero

**Affiliations:** 1 Universidad de Manizales, Manizales, Caldas, Colombia

The integration of non-pharmacological approaches with smart-environment technologies, combined with a mixed-methods design and the development of a practical methodological guide, represents a meaningful and potentially impactful contribution (particularly for Latin American health systems where caregiving is largely informal and resource constraints are significant).

 -From a conceptual standpoint, the manuscript successfully describes the intersection between technology, caregiving, and local context. However, its broader contribution to health systems and implementation science remains somewhat implicit. At present, the study reads primarily as a contextual needs assessment rather than as a scalable or transferable model. I would strongly encourage the authors to explicitly frame their work within the domain of implementation-oriented digital health. For instance, the final paragraph of the introduction could be strengthened with a statement such as:

 “Beyond addressing a local care need, this study contributes to the emerging field of implementation-oriented digital health in low- and middle-income settings. Specifically, it reframes smart-environment interventions not merely as assistive tools, but as components of scalable, context-sensitive care models that can be embedded within fragmented health systems such as those observed in Latin America.”

 -Methodologically, the study is coherent and appropriately designed as an exploratory mixed-methods analysis. However, there is a noticeable tension between the formulation of hypotheses (H1–H3), which imply explanatory or even causal relationships, and the actual analytical capacity of the study (cross-sectional design, small sample size, and highly unbalanced subgroups, particularly the patient group, n=3). While this does not invalidate the study, it does require a more cautious framing of the findings. I recommend explicitly acknowledging this in the Methods or early Results section. A possible wording would be:

 “Given the exploratory nature of the design and the limited sample size (particularly within patient subgroups) hypothesis testing should be interpreted as indicative rather than confirmatory. Accordingly, the analyses are framed as hypothesis-generating, with emphasis placed on effect sizes and patterns rather than statistical significance alone.”

 -In addition, although content validity of the instrument is reported through expert judgment, the absence of formal psychometric validation should be transparently acknowledged. This can be incorporated into the limitations section as follows:

 “Although content validity was established through expert judgment, the absence of formal psychometric validation (e.g., construct validity, internal consistency, test-retest reliability) may affect measurement precision and comparability across settings.”

 -The most critical issue, in my view, relates to the interpretation of results. In several sections (particularly within the Discussion), the manuscript suggests improvements in quality of life, autonomy, and well-being. However, these outcomes were not directly measured using validated instruments, nor assessed longitudinally. This creates a risk of overinterpretation. A more cautious and precise wording would substantially strengthen the manuscript without weakening its contribution. For example:

 “Importantly, while the findings suggest patterns consistent with improved autonomy, safety, and perceived well-being, these outcomes were not directly measured through validated longitudinal instruments. Therefore, interpretations regarding quality of life improvements should be considered inferential and hypothesis-generating rather than demonstrative.”

 -Similarly, toward the end of the Discussion, the authors may consider reframing the scope of their contribution:

 “Rather than demonstrating causal effects, this study identifies a structured alignment between user-perceived needs and the functional affordances of low-burden smart technologies, providing a foundation for future implementation trials.”

 -From a health services perspective, the manuscript presents an excellent opportunity that is not yet fully leveraged. While the legal and policy framework in Colombia is mentioned, the implications for health system organization, scalability, and service delivery models remain underdeveloped. Strengthening this dimension would significantly increase the manuscript’s impact. A possible addition in the implications section could be:

 “From a health systems perspective, the proposed model may serve as a micro-level innovation with potential macro-level implications. In fragmented health systems, such as Colombia’s, smart-environment-based care could act as a decentralized extension of formal services, reducing pressure on institutional care while improving continuity of care at the household level.”

 -Finally, regarding writing style, the manuscript is clear and technically sound, but at times it reflects a highly uniform and overly explicit logical structure. Introducing subtle variation in sentence construction and allowing some degree of implicit reasoning would improve its readability and align it more closely with mature academic English. For instance, a sentence such as:

 “Taken together, these results suggest that who the participant is in the care network better explains patterns of exposure and use than age or sex alone”

 could be refined to:

 “What appears to matter most is not age or sex per se, but the position each individual occupies within the care network. Patterns of use seem to emerge from these relational roles rather than from demographic attributes alone.”

Is the work clearly and accurately presented and does it cite the current literature?

Yes

If applicable, is the statistical analysis and its interpretation appropriate?

Yes

Are all the source data underlying the results available to ensure full reproducibility?

Yes

Is the study design appropriate and is the work technically sound?

Yes

Are the conclusions drawn adequately supported by the results?

Partly

Are sufficient details of methods and analysis provided to allow replication by others?

Yes

Reviewer Expertise:

Clinical Medicina, Biomedical Sciences, Scientific Writing.

I confirm that I have read this submission and believe that I have an appropriate level of expertise to confirm that it is of an acceptable scientific standard.
